# Hydrostatic Pressure Affects *In Vitro* Maturation of Oocytes and Follicles and Increases Granulosa Cell Death

**Published:** 2013-11-20

**Authors:** Zahra Rashidi, Mehri Azadbakht, Ali Amini, Isac Karimi

**Affiliations:** 1 Department of Biology, Faculty of Basic Sciences, Razi University, Kermanshah, Iran; 2 Department of Basic Sciences, College of Veterinary Medicine, Razi University, Kermanshah, Iran

**Keywords:** *In vitro* Maturation, Oocyte, Hydrostatic Pressure, Apoptosis, Mouse

## Abstract

**Objective:**

This study examines the effects of hydrostatic pressure on *in vitro* maturation (IVM) of oocytes derived from *in vitro* grown follicles.

**Materials and Methods:**

In this experimental study, preantral follicles were isolated from 12-day-old female NMRI mice. Each follicle was cultured individually in Alpha Minimal Essential Medium (α-MEM) under mineral oil for 12 days. Then, follicles were induced for IVM and divided into two groups, control and experiment. In the experiment group follicles were subjected to 20 mmHg pressure for 30 minutes and cultured for 24-48 hours. We assessed for viability and IVM of the oocytes. The percentage of apoptosis in cumulus cells was determined by the TUNEL assay. A comparison between groups was made using the student’s t test.

**Results:**

The percentage of metaphase II oocytes (MII) increased in hydrostatic pressuretreated follicles compared to controls (p<0.05). Cumulus cell viability reduced in hydrostatic pressure-treated follicles compared to controls (p<0.05). Exposure of follicles to pressure increased apoptosis in cumulus cells compared to controls (p<0.05).

**Conclusion:**

Hydrostatic pressure, by inducing apoptosis in cumulus cells, participates in the cumulus oocyte coupled relationship with oocyte maturation.

## Introduction

*In vitro* maturation (IVM) of mammalian oocytes is an efficient method to produce mature oocytes for their use in assisted reproductive techniques (1). Induction of ovulation to obtain mature oocytes for *in vitro* fertilization (IVF) is a routine procedure in numerous infertility clinics. Some women, however, may fail to respond to hormonal stimulation or are at risk of ovarian hyperstimulation (2). IVM of oocytes offers an alternative strategy to obtain mature oocytes in these cases (3,4). The fertility rate from matured oocytes *in vitro* is much lower than those of *in vivo* stimulation cycles, indicating that improvement in IVM remains a challenge (5).

Supplementation of the media with hormones (6), growth factors (7), optimization of culture systems (8,9), and environmental and physical conditions of follicles (10) have been proposed to increase quality of IVM oocytes. In this sense, the later factors have an important role in the success of IVM. Physical forces affect follicle rupture and ovulation by increasing follicular fluid pressure due to an increase in hydrostatic pressure in the ovarian vascular system. A decrease of tensile strength in the follicle wall and increase of the hydrostatic pressure inside the follicle, or a combination of these events is needed for successful follicular rupture (11). Physical forces may cause tissue thinning and follicular rupture by elimination of selective cumulus cells (12). On the other hand, cumulus cells dissociate during the ovulatory process and the oocyte is freed into follicular fluid. Programmed cell death participates in degeneration of follicular cells, weakens the follicular wall and ruptures. The amount of cell death in the cumulus oocyte complex (COCs) that impacts oocyte development potential is unclear (13). Development of follicles and their related COCs is influenced by various apoptotic mechanisms (14). The spatiotemporal pattern of apoptosis during follicle growth and oocyte maturation is tightly regulated (11). Disruption of either timing or the magnitude of apoptosis can alter cell connectivity in the cumulus mass and between cumulus cells and the oocyte, causing deficits in oocyte quality. The degree of apoptosis is correlated with developmental competence of the enclosed oocytes (15). It has been suggested that moderate apoptotic changes in the follicle may support or induce prematuration-like changes of the oocyte which is typical for their preovulatory development (16).

Hydrostatic pressure is the pressure exerted by a static fluid that depends on the fluid’s depth, density and gravity. It independent of shape, total mass or surface area of the fluid (17). Hydrostatic pressure, as a physical force, plays various physiological roles in the reproductive system. Increased intrafollicular pressure and spontaneous contractions, together with the enzymatic degradation of the extracellular matrix, may be important for rupture of the follicle at ovulation (11). The main finding of previous studies is the presence of a relatively constant intrafollicular pressure, between 15 and 20 mmHg, during the entire ovulatory process (18-20). Hydrostatic pressure has been demonstrated to induce cell death in different cell types (21,22). In a previous study we have shown that hydrostatic pressure enhanced the IVM of the oocytes from non-vitrified and vitrified-warmed ovaries and increased the incidence of cell death in cumulus cells without a sign of cell death in mouse oocytes (23). We took into consideration the exposure of COCs to intrafollicular pressure in the ovulating follicles during the late ovulatory process and hydrostatic pressure as a cell death inducer. Thus, we designed the present study to investigate the effect of hydrostatic pressure on inducing cell death in COCs and to improve oocyte IVM of oocytes derived from in vitro grown follicles. In the present study, hydrostatic pressure was used to investigate the involvement of COC cell death on the IVM of oocytes derived from *in vitro* grown follicles.

## Materials and Methods

Chemicals were purchased from Sigma Chemical Co. (St. Louis, MO, USA) and Gibco (Grand Island, NY, USA) unless otherwise indicated.

### Ovarian follicle recovery and culture

This experimental study was reviewed and approved by the Laboratory Animal Care Committee of the Faculty of Basic Sciences, Razi University, Kermanshah, Iran. In this study, 12- 14 day-old female NMRI mice were prepared from Razi Vaccine and Serum Research Institute, Iran. Animals were maintained at a temperature of 20-25˚C and 50% humidity under light-controlled conditions (12 hours light: 12 hours dark) and provided with food and water ad libitum.

After the mice were sacrificed, their ovaries were removed and immediately transferred to dissection medium that consisted of Alpha Minimal Essential Medium (α-MEM), supplemented with 10% fetal bovine serum (FBS), 100 IU/ml penicillin G, and 100 μg/ml streptomycin. Follicles were isolated by mechanical dissection under a stereomicroscope (Motic: SMZ-143, China) at 10x magnification, using 27 G sterile needles to ensure that the follicular structure remained intact. Follicles were selected according to the following criteria: i. intact follicles with one or two layers of granulosa cells and some adhering theca cells; ii. visible, round and central oocyte, and iii. follicle diameter between 120-140 μm. Follicles were rinsed three times in dissection medium and transferred to culture medium that consisted of dissection medium supplemented with 100 mIU/ml recombinant follicle stimulating hormone (rFSH, Gonal-F; Sereno, Inc) and 10 ng/ml recombinant epidermal growth factor (rEGF). Follicles were cultured according to a previously described method with some modifications (24). Briefly, follicles were cultured individually in 20 μl microdrops under detoxified mineral oil in a 60 mm tissue culture plate (Falcon; France) at 37˚C. Under an atmosphere of 5% CO_2_ in air for 12 days. In addition, the medium was equilibrated overnight prior to the start of culturing. At every 48 hours of culture, we replaced 10 μl of the culture medium from each drop with fresh medium.

### Follicle monitoring and measurement

During the culture period follicles were monitored daily under an inverted microscope (Olympus, Japan). Follicle and oocyte quantitative measurement of morphological features were performed according to a previously described method with modifications (24). For each follicle, two perpendicular diameters were measured using a calibrated ocular micrometer (Dino Digital Eyepiece: AM323, Taiwan), at a magnification of ×200, before culture, and then on days 3, 6, 9 and 12 of culture. Follicle diameter was recorded in micrometers. Spindle shaped theca cells which originated from the follicle theca and attached to the dish were not included in the measurements. In addition, the same measurement for each oocyte was calculated. After the oocytes were retrieved from the follicles, we recorded their diameters (from the outer layer of the zone on one side to the outer layer of the zone on the opposite side), along with the longest length and widest perpendicular width.

### Experimental protocol

On day 12 we chose good quality follicles which were defined as intact follicles with a central oocyte surrounded by a granulosa cell mass, peripheral spindle-shaped theca cell monolayer, visible, round and central oocyte, and follicle diameter ≥500 μm. Follicles were allocated and placed in culture medium randomly and divided into two groups, experiment and control. In the experiment group, follicles were transferred to a pressure chamber according to an established model (21), after which they were subjected to 20 mmHg hydrostatic pressure for 30 minutes. Follicles in the control group were transferred to a similar pressure chamber for 30 minutes, but were not exposed to any hydrostatic pressure. After depressurization, the culture plates were removed from the pressure chamber and cultured for 24-48 hours. After 24-48 hours, we assessed the IVM of oocytes derived from *in vitro* grown follicles as well as the cell death incidence and apoptosis in the cumulus oocyte complexes (COCs). The experiment was repeated at least nine times for evaluation of IVM and five times for assessment of cell death and apoptosis.

### *In vitro* maturation of oocytes

IVM was performed according to a previously described method with some modifications (24). Follicles in both groups were cultured in culture medium supplemented with 5 IU/ml human chorionic gonadotropin (hCG; Sereno, Inc). After 24 and 48 hours in culture, we evaluated the oocytes for nuclear maturation and viability.

### Evaluation of nuclear maturation

To assess the rate of meiosis at the end of the maturation period, follicles were mechanically ruptured, oocytes were denuded and their nuclear maturation status assessed to observe for the presence of germinal vesicles (GV), germinal vesicle breakdown (GVBD), metaphase II (MII) and parthenogenetic embryos (PA) under an inverted microscope (Olympus IX 71; Japan).

### Oocyte viability

In this assessment the follicles were mechanically ruptured and the oocytes were denuded, after which their viability was assessed. Oocytes were incubated in 500 μl of 50 μg/ml propidium iodide (PI) in α-MEM for 30 seconds. Oocytes were rinsed in PBS and observed under inverted microscope equipped with an ultraviolet lamp at 560 nm (Olympus, Japan).

### Hoechst/propidium iodide nuclear staining of cumulus oocyte complexes

Hoechst/propidium iodide (PI) nuclear staining is routinely used for quantitative analysis of cell death. Supravital nuclear staining of COCs was performed according to the method described previously with slight modifications (25). Briefly, at 24 hours after IVM the COCs were incubated with the cell-permeant dye bisbenzamide (Hoechst 33258; 10 μg/ ml in α-MEM) for 15 minutes at 37˚C. Next, cells were washed and immediately transferred into the cell-impermeant dye, PI (50 μg/ml in α-MEM) just before microscopy. Stained COCs were subsequently mounted in glycerol, gently flattened with a coverslip and visualized for cell counting on a fluorescence microscope (Olympus, AX70; Japan) with excitation filters at 460 nm for blue and red fluorescence.

### Terminal deoxy-nucleotidyl transferase-mediated (dUTP) nick-end labeling (TUNEL)

The terminal deoxy-nucleotidyl transferasemediated (dUTP) nick-end labeling (TUNEL) procedure was used to detect DNA fragmentation in combination with PI counterstaining to assess nuclear morphology. Nuclear DNA fragmentation in COCs was detected by the TUNEL method using an In Situ Cell Death Detection Kit (Roche Diagnostics Corporation Mannheim, Germany). The method was previously described in detail (26). Briefly, 24 hours after IVM COCs were removed from culture medium, washed three times in PBS that contained 1 mg/ ml PVP, fixed in 4% (w/v) paraformaldehyde in PBS for 1 hour at room temperature, and stored in PBS-PVP at 4˚C. Then, COCs were permeabilized in 100 μl drops of 0.1% (v/v) Triton X-100 that contained 0.1% (w/v) Na-citrate in PBS for 30 minutes at room temperature. Next, COCs were washed three times in PBS. The COCs were placed in 30 μl drops of TUNEL reagent that contained ﬂuorescein isothiocyanate conjugated dUTP and the enzyme terminal deoxy-nucleotidyl transferase (as prepared by the manufacturer), then incubated in the dark for 1 hour at 37˚C in a humidified chamber. The negative controls were incubated in the absence of terminal deoxy-nucleotidyl transferase. The positive control was incubated with 30000 U/ ml DNaseI solution for 10 minutes at 37˚C, then rinsed with PBS. COCs were washed in PBS-PVP and transferred to 100 μl drops of 50 μg/ml PI in PBS-PVP for 30 minutes in a dark chamber at room temperature. The COCs were washed four times in PBS-PVP to remove excess PI, then they were mounted in glycerol onto a slide and placed under a cover slip. The COCs were observed under a ﬂuorescent microscope (Olympus AX70; Japan). The apoptotic index of the COCs was calculated as the percentage of apoptotic cells relative to the total cell number.

### Statistical analysis

Data for oocyte viability and IVM, the means of COC cell death and apoptotic index in the COCs were analyzed by the student’s t test. For the statistical analysis we utilized SPSS version 16 software. Data were expressed as mean ± SEM and p<0.05 was considered as a minimum criterion for assigning statistical significance.

## Results

### Follicle and oocyte measurement

**Fig 1:**
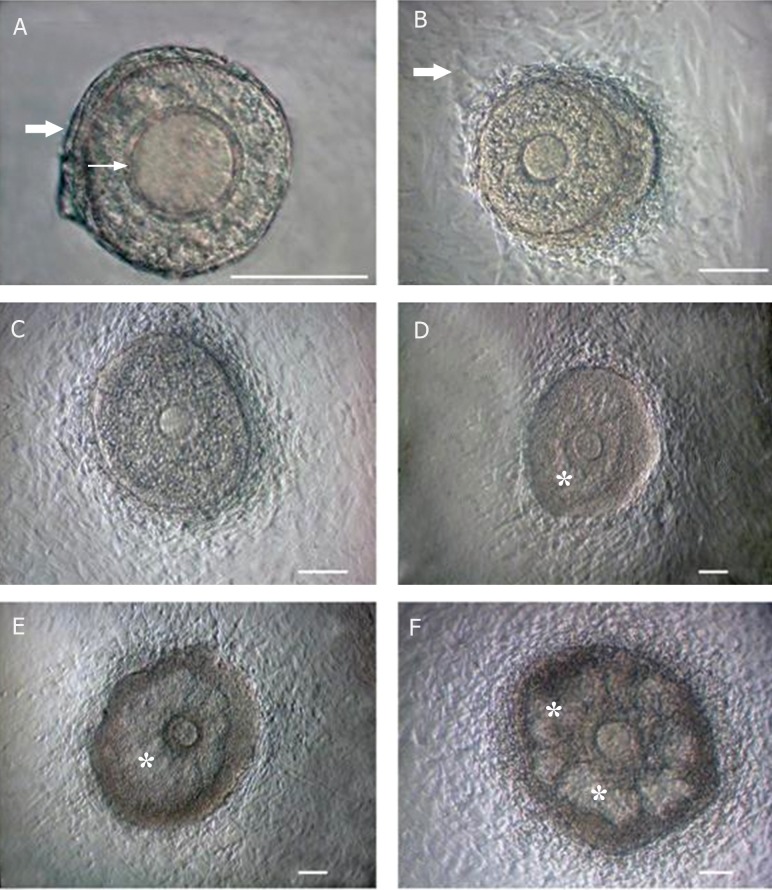
Morphology of mouse follicle during *in vitro* growth. The cultured isolated follicle on day 1 (A), day 3 (B), day 6 (C), day 9 (D) and day 12 (E, F). **→** ; Theca cells monolayer, → ; Zona pellucid and *; Antral cavity. Scale bar: 100 μm.

Preantral follicular diameter increased during in vitro culture. Granulosa and thecal cell outgrowth were prominent and the antral cavities were visualized as clear cavities in follicles from day 9 (Fig 1). The diameters of the cultured follicles at different days of culture (1, 3, 6, 9 and 12) are shown in table 1 (p<0.05).

**Table 1:** Follicle and oocyte diameters during culture


**Follicle diameter**	54	133.02 ± 2.05	158.77 ± 3.98	256.77 ± 11.72	359.75 ± 22.3	494.31 ± 24.05
**Oocyte diameter**	54	47.33 ± 0.41	52.05 ± 1.51	64.25 ± 1.43	70.32 ± 0.75	72.29 ± 0.58


Two perpendiculars were measured at ×200 magnification and is calculated based on micrometers. Mean diameters ± SEM were calculated.

### Viability and *in vitro* maturation of oocytes

At 0 and 24 hours, viability of the oocytes was similar between the experiment and control groups. At 24 hours, hydrostatic pressure did not significantly alter the percentage of GV oocytes, whereas the percentage of GVBD and MII oocytes increased in the experiment group compared to the control group (p<0.05). At 48 hours, the percentage of GVBD oocytes was similar between the experiment and control groups, while the percentage of GV oocytes decreased in the experiment group compared to the control group (p<0.05). The percentages of MII oocytes and PA embryos increased in the experiment group compared to the control group (p<0.05; Table 2).

### Nuclear staining of the cumulus oocyte complexes

**Fig 2:**
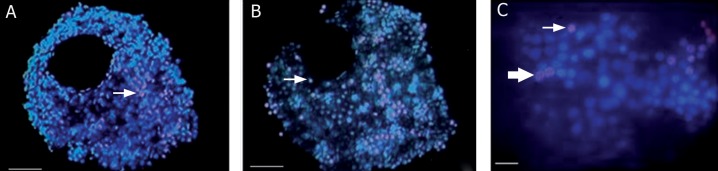
Cell death in COCs as determined by fluorescence microscopy after 24 hours of pressure exposure. A. Control group; without pressure exposure. B. Experiment group; Exposure to pressure. Viable cells; Blue-stained smooth nuclei or multiple bright specks of condensed chromatin, Dead cells; Pink-stained nuclei with multiple bright specks of fragmented chromatin or more spheres of condensed chromatin and →; Dead cells. C. Characteristics of viable and dead cell, **→**; Fragmented nucleus and →; Condensed nucleus. Scale Bar: A, B 50 μm and C: 10 μm.

The percentage of viable cells was lower in COCs from the experiment group compared to the control at 0 and 24 hours of hydrostatic exposure (p<0.05). The percentages of fragmented and condensed nuclear cumulus cells in non-viable cells were higher in the experimental group compared to the control group (p<0.05; Table 3). Cell morphology was scored as follows. Viable cells contained blue-stained normal, smooth nuclei or multiple bright specks of chromatin. Non-viable cells consisted of pink-stained nuclei with either multiple bright specks of fragmented chromatin which included discrete clusters of membrane-bounded vesicles and one or more spheres of condensed chromatin (significantly more compact and smaller than normal nuclei) as seen in figure 2.

**Table 2:** Viability and IVM of oocytes derived from *in vitro* grown follicles


			24 hours				48 hours			
**Groups**	N	Viability	GV	GVBD	MII	PA	GV	GVBD	MII	PA

**Control**	89	100%	32	48	8	1	22	44	18	5
			(35.9%)^a^	(53.9%)^a^	(9.1%)^a^	(1.1%)^a^	(24.7%)^a^	(45.8%)^a^	(20.2%)^a^	(5.6%)^a^
**Experiment**	96	100%	29	42	15	9	12	40	32	12
			(30.2%)^a^	(43.7%)^b^	(15.6%)^b^	(10.4%)^b^	(12.5%)^b^	(41.7%)^a^	(33.3%)^b^	(12.5%)^b^


Control group; No pressure exposure and experiment group; Exposure to pressure. GV; Germinal vesicle, GVB; Germinal vesicle breakdown, MII; Metaphase II and PA; Parthenogenetic embryo. Different superscripts indicate significant differences (p<0.05).

**Table 3:** Cell death in COCs derived from *in vitro* grown follicles


			0 hour			24 hours		
Groups	N	Total cells	Viable cells	Non-viable cells	Total cells	Viable cells	Non-viable cells

**Control**	25	756.5 ± 61.9 ^a^	737	4.3	13.8	404.4 ± 26.4 ^a^	375
			(97.3%)^a^	(0.66%)^a^	(1.89%)^a^		(93.2%)^a^
**Experiment**	25	748.7 ± 65.3 ^a^	628	14.5	31.6	391.9 ± 24.6 ^a^	315
			(93.5%)^b^	(2.02%)^b^	(4.42%)^b^		(85.5%)^b^


Control group; No pressure exposure and experiment group; Exposure to pressure. Different superscripts indicate significant differences (p<0.05).

### TUNEL labeling

**Fig 3:**
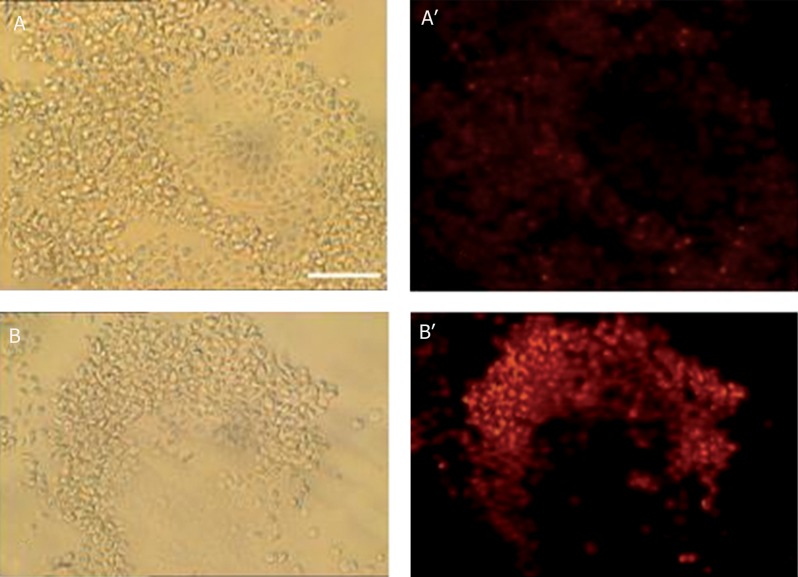
Identification of apoptosis in COCs following TUNEL staining and counterstaining with propidium iodide (PI) after 24 hours of pressure exposure Apoptotic nuclei identified by the observation of a distinct bright yellow stained chromatin. A. Phase contrast picture from control group. A'. Match figure TUNEL staining by fluorescence microscopy in control group that had no pressure exposure. B. Phase contrast picture from experiment group exposed to pressure. B'. Math figure TUNEL staining by fluorescence microscopy in experiment group exposed to pressure. Scale bar: 50 μm.

The apoptotic index was higher in COCs from the experiment group compared with the control group at 0 and 24 hours of hydrostatic exposure (p<0.05; Table 4). TUNEL reaction was assessed by the observation of a distinct bright yellow stained chromatin. Nuclear morphology was assessed on the basis of PI staining. The nuclei were classiﬁed according to four clear types of morphology: healthy interphase nuclei with uniform PI staining and a clear outline; mitosis, which included cells at the prophase, metaphase or anaphase stages with visible chromosomes counted as single nuclei; fragmented nuclei, which included discrete clusters of membrane-bounded vesicles; and condensed nuclei with intense PI staining, which were smaller than 'healthy' interphase nuclei. According to the above criteria, the nuclei that displayed morphological characteristics of apoptosis (condensation and fragmentation) and biochemical characteristics of apoptosis (TUNEL reaction positive) were considered to be apoptotic nuclei (Fig 3).

**Table 4:** Apoptosis in COCs derived from *in vitro* grown follicles


		0 hour		24 hours	
Groups	N	Total cells	Apoptotic index	Total cells	Apoptotic index

**Control**	25	764.6 ± 60.2 ^a^	11.2 (2.4%)^a^	416.2 ± 26.4 ^a^	21.4 (4.5%)^a^
**Experiment**	25	759.7 ± 62.5 ^a^	22.2 (4.7%)^b^	409.6 ± 24.6 ^a^	30.1 (6.7%)^b^


Control group; No pressure exposure and experiment group; Exposure to pressure. Different superscripts indicate significant difference (p<0.05).

## Discussion

The present study indicated that the IVM rate in oocytes derived from preovulatory follicles in vitro increased following exposure to hydrostatic pressure. The hydrostatic pressure increased the mild cell death in cumulus cells in experimental group without any adverse effects on the survival rate of oocytes. The viability of oocytes derived from both groups was similar and independent of exposure to hydrostatic pressure. The percentage of parthenogenesis increased in oocytes exposed to hydrostatic pressure.

Folliculogenesis and meiotic maturation are time dependent processes (27). Previously, different culture systems have been evaluated for a narrow class of intact preantral follicles retrieved from mice (28). Our culture system is based upon the liquid-phase model as an open culture system (27,29). Nutrients, hormones and gases are more available in an open system than in a closed system, which increases oocyte survival rate. Follicles that have been cultured *in vitro* for 12 days are equivalent to antral follicles in 24-day-old mouse ovaries. This time-point corresponds to the first wave of meiotic maturation leading to ovulation (24).

In the current experiment, preantral follicles isolated from ovarian tissue grew *in vitro* between days 1 and 12. Follicle growth slowed slightly after day 10, but oocytes continued to develop and reached a diameter similar to that of fully grown oocytes *in vivo*.

Researchers have successfully achieved IVM of preantral follicle-enclosed oocytes and of oocytegranulosa- cell complexes from preantral follicles obtained from mouse ovaries (24,30). Physiologically, this maturation process is dependent upon several biotical and abiotical parameters. Numerous experiments have been performed to optimize *in vitro* conditions, which should imitate *in vivo* conditions (31). In these circumstances, abiotic parameters consist of temperature (32), pH (33), and osmotic and hydrostatic pressures (34,35). The most important biotic parameters are organic substances in media, hormones (36), and activators and inhibitors of IVM (31). Hydrostatic pressure, in contrast to other parameters, acts immediately and uniformly at each point of the *in vitro* production (IVP). It can be applied with the highest precision, consistency, and reliability to mimic *in vivo* conditions. It has been reported that a well-defined sub-lethal high hydrostatic pressure treatment offers a solution to improve the overall quality of gametes and embryos, fertilizing ability, and developmental competence (37). Du et al. (35) have shown that pre-treatment with a high hydrostatic pressure considerably improved the IVP of porcine vitrified oocytes.

Matousek et al. have reported an increase in intrafollicular pressure during the ovulating process (11). A basal intrafollicular pressure of 16.6 ± 1.0 mmHg was reported at the preovulatory phase (48 hours after eCG) which increased gradually throughout the ovulatory process to 21.4 ± 2.4 mmHg at 4-7 hours after hCG (midovulatory phase) and 23.9 ± 1.9 mmHg at 8-12 hours after hCG. The intrafollicular pressures have been measured in the preovulatory follicles of cows (18), hamsters (19) and rabbits (20). These measurements were obtained by inserting a large micropipette into the follicular antrum after which the passive intrafollicular pressure was recorded. The main finding of these studies showed that COCs were exposed to intrafollicular pressures between 15-20 mmHg during the entire ovulatory process (11). In a previous study, we reported that 20 mmHg of hydrostatic pressure induced mild cell death in cumulus cells, decreased cell junctions and waste paracrine correlation between cumulus cells and oocytes, and induced maturation of oocytes derived from vitrified-warmed mouse ovaries (23).

According to the results of the above mentioned observations, we selected a pressure of 20 mmHg for the present investigation. The percentage of MII oocytes considered as oocyte maturation significantly increased in follicles exposed to hydrostatic pressure compared to those that unexposed to hydrostatic pressure. These results indicated that hydrostatic pressure improved oocyte maturation. Concomitantly with improved oocyte maturation, hydrostatic pressure increased cell death in COCs derived from preovulatory follicles *in vitro*. Hydrostatic pressure increased cell death in cumulus cells, which have a critical role in oocyte maturation and fertilization. On the other hand, cumulus cells dissociate during the ovulatory process, releasing the oocyte into the follicular fluid antrum (13).

A study by Ikeda et al. (15) has demonstrated that cumulus cells in bovine cumulus-enclosed oocytes spontaneously underwent apoptosis during IVM. Apoptotic changes in the follicle possibly support or induce prematuration-like changes to the oocyte which is typical for their preovulatory development (16). Cumulus cells play an important role in oocyte maturation by keeping the oocyte under meiotic arrest, inducing meiotic resumption and supporting cytoplasmic maturation (1). Cumulus cells and oocytes have a relationship in preovulatory follicles that due to paracrine and regulation factors convenience available for oocyte. The signals that produced by cumulus cells even with waste gap junction cumulus cells affected on maturation of oocyte (38,39).

In the dead cells, some of the nuclei were fragmented and condensed. The percentage of these types of nuclei in cumulus cells were increased in hydrostatic pressure-treated follicles. Fragmentation and condensation of nuclei are two morphological features of apoptotic cells, therefore in the current study, the type of cell death observed in cumulus cells was apoptosis, as confirmed by TUNEL staining. Investigation of apoptotic cell death by TUNEL staining has been performed in previous studies (26,40). The percentage of TUNEL-positive cells was considered to be apoptotic cells that significantly increased in follicles exposed to hydrostatic pressure. Hydrostatic pressure as a cell death inducer (21,22) with increasing apoptotic cells in COCs led to increasing oocyte maturation compared with the group that had no exposure to hydrostatic pressure.

Hydrostatic pressure increased the percentage of parthenogenetic oocytes, as reported in previous studies (35,41). Our data indicated that the percentage of parthenogenetic oocytes significantly increased in follicles exposed to hydrostatic pressure compared to unexposed follicles.

Hydrostatic pressure caused an increase in the rate of cumulus cells that underwent apoptosis and probably be responsible for the increased MII oocyte rate after IVM.

There is increasing evidence that hydrostatic pressure plays important roles in cell shape and structure, exocytosis, and growth and death of animal cells. Although reproductive biology has been dominated by a focus on genes and chemical interactions over the past century, it is time to further explore the mechanism by which mechanical forces can exert their potent effects on gametes and embryos during reproduction, as well as throughout adult life (42). Pro-apoptotic effects of hydrostatic pressure and the pivotal role of apoptosis in ovulation prompt us to investigate the effects of hydrostatic pressure on the IVM of mouse oocytes and on apoptosis in COCs from ovarian follicles.

## Conclusion

This study implicitly explains a model system to develop an understanding of the link between the physical condition of a follicle and the ovulatory process. According to the results of this study, hydrostatic pressure can be used to increase the apoptosis rate of cumulus cells; the latter may be responsible for an increase in the MII oocyte rate after IVM. We have shown that hydrostatic pressure had a mild effect on the incidence of cell death in cumulus cells but no aberrant effect on oocyte viability.

## References

[1] Mahmoudi R, Subhani A, Pasbakhsh P, Abolhasani F, Amiri I, Salehnia M (2005). The effects of cumulus cells on in vitro maturation of mouse germinal vesicle stage oocytes. Iran J Reprod Med.

[2] Child TJ, Abdul-Jalil AK, Gulekli B, Tan SL (2001). In vitro maturation and fertilization of oocytes from unstimulated normal ovaries, polycystic ovaries, and women with polycystic ovary syndrome. Fertil Steril.

[3] Tavana S, Eimani H, Azarnia M, Shahverdi A, Eftekhari- Yazdi P (2012). Effects of Saffron (Crocus sativus L.) Aqueous Extract on In vitro Maturation, Fertilization and Embryo Development of Mouse Oocytes. Cell J.

[4] Hardy K, Wright CS, Franks S, Winston RM (2000). In vitro maturation of oocytes. Br Med Bull.

[5] Bos-Mikich A, Ferreira M, Höher M, Frantz G, Oliveira N, Dutra CG (2011). Fertilization outcome, embryo development and birth after unstimulated IVM. J Assist Reprod Genet.

[6] Cortvrindt R, Smitz J, Van Steirteghem AC (1997). Assessment of the need for follicle stimulating hormone in early preantral mouse follicle culture in vitro. Hum Reprod.

[7] Mao J, Smith MF, Rucker EB, Wu GM, McCauley TC, Cantley TC (2004). Effect of epidermal growth factor and insulin-like growth factor I on porcine preantral follicular growth, antrum formation, and stimulation of granulosal cell proliferation and suppression of apoptosis in vitro. J Anim Sci.

[8] Itoh T, Hoshi H (2000). Efficient isolation and long-term viability of bovine small preantral follicles in vitro. In Vitro Cell Dev Biol Anim.

[9] Telfer EE, McLaughlin M, Ding C, Thong KJ (2008). A twostep serum-free culture system supports development of human oocytes from primordial follicles in the presence of activin. Hum Reprod.

[10] Sirotkin AV (2010). Effect of two types of stress (heat shock/ high temperature and malnutrition/serum deprivation) on porcine ovarian cell functions and their response to hormones. J Exp Biol.

[11] Matousek M, Carati C, Gannon B, Brännström M (2001). Novel method for intrafollicular pressure measurements in the rat ovary: increased intrafollicular pressure after hCG stimulation. Reproduction.

[12] Murdoch WJ (1995). Programmed cell death in preovulatory ovine follicles. Biol Reprod.

[13] Murdoch WJ, Gottsch ML (2003). Proteolytic mechanisms in the ovulatory folliculo-luteal transformation. Connect Tissue Res.

[14] Tilly JL (1996). Apoptosis and ovarian function. Rev Reprod.

[15] Ikeda S, Imai H, Yamada M (2003). Apoptosis in cumulus cells during in vitro maturation of bovine cumulusenclosed oocytes. Reproduction.

[16] Hendriksen PJ, Vos PL, Steenweg WN, Bevers MM, Dieleman SJ (2000). Bovine follicular development and its effect on the in vitro competence of oocytes. Theriogenology.

[17] Zonia L, Munnik T (2007). Life under pressure: hydrostatic pressure in cell growth and function. Trends Plant Sci.

[18] Bronson RA, Bryant G, Balk MW, Emanuele N (1979). Intrafollicular pressure within preovulatory follicles of the pig. Fertil Steril.

[19] Talbot P (1983). Intrafollicular pressure promotes partial evacuation of the antrum during hamster ovulation in vitro. J Exp Zool.

[20] Espey LL, Lipner H (1963). Measurements of intrafollicular pressures in the rabbit ovary. Am Physiol.

[21] Agar A, Yip SS, Hill MA, Coroneo MT (2000). Pressure related apoptosis in neuronal cell lines. J Neurosci Res.

[22] Agar A, Li S, Agarwal N, Coroneo MT, Hill MA (2006). Retinal ganglion cell line apoptosis induced by hydrostatic pressure. Brain Res.

[23] Rashidi Z, Azadbakht M, Khazaei M (2012). Hydrostatic pressure improves in-vitro maturation of oocytes derived from vitrified-warmed mouse ovaries. Iran J Reprod Med.

[24] Pesty A, Miyara F, Debey P, Lefevre B, Poirot C (2007). Multiparameter assessment of mouse oogenesis during follicular growth in vitro. Mol Hum Reprod.

[25] Shacter E, Williams JA, Hinson RM, Sentürker S, Lee YJ (2000). Oxidative stress interferes with cancer chemotherapy: inhibition of lymphoma cell apoptosis and phagocytosis. Blood.

[26] Pocar P, Nestler D, Risch M, Fischer B (2005). Apoptosis in bovine cumulus-oocyte complexes after exposure to polychlorinated biphenyl mixtures during in vitro maturation. Reproduction.

[27] Smitz JE, Cortvrindt RG (2002). The earliest stages of folliculogenesis in vitro. Reproduction.

[28] Martins OG, Pesty A, Gouveia-Oliveira A, Cidadão AJ, Plancha CE, Lefevre B (2002). Oocyte Ca2+ spike acquisition during in vitro development of early preantral follicles: influence of age andhormonal supplementation. Zygote.

[29] Cortvrindt R, Smitz J, Van Steirteghem AC (1996). In-vitro maturation, fertilization and embryo development of immature oocytes from early preantral follicles from prepuberal mice in a simplified culture system. Hum Reprod.

[30] Demeestere I, Delbaere A, Gervy C, Van Den Bergh M, Devreker F, Englert Y (2002). Effect of preantral follicle isolation technique on in-vitro follicular growth, oocyte maturation and embryo development in mice. Hum Reprod.

[31] Smiljaković T, Sretenović Lj, Aleksić S (2009). Influence of abiotic and biotic factors on maturation of oocytes (mammalian eggs) in vitro conditions. Biotech Anim Husbandry.

[32] Sugiyama S, McGowan M, Phillips N, Kafi M, Young M (2007). Effects of increased ambient temperature during IVM and/or IVF on the in vitro development of bovine zygotes. Reprod Domest Anim.

[33] Smiljaković T, Josipovic S, Kosovac O, Delic N, Aleksic S, Petrovic MM (2008). The role of pH values in porcine reproductive tracts of male and female individuals. Biotech Anim Husbandry.

[34] Van den Abbeel E, Schneider U, Liu J, Agca Y, Critser JK, Van Steirteghem A (2007). Osmotic responses and tolerance limits to changes in external osmolalities, and oolemma permeability characteristics, of human in vitro matured MII oocytes. Hum Reprod.

[35] Du Y, Pribenszky CS, Molnár M, Zhang X, Yang H, Kuwayama M (2008). High hydrostatic pressure: a new way to improve in vitro developmental competence of porcine matured oocytes after vitrification. Reproduction.

[36] Reinthaller A, Kirchheimer JC, Deutinger J, Bieglmayer C, Christ G, Binder BR (1990). Plasminogen activators, plasminogen activator inhibitor, and fibronectin in human granulosa cells and follicular fluid related to oocyte maturation and intrafollicular gonadotropin levels. Fertil Steril.

[37] Pribenszky C, Du Y, Molnár M, Harnos A, Vajta G (2008). Increased stress tolerance of matured pig oocytes after high hydrostatic pressure treatment. Anim Reprod Sci.

[38] Kawamura K, Kawamura N, Mulders SM, Sollewijn Gelpke MD, Hsueh AJ (2005). Ovarian brain-derived neurotrophic factor (BDNF) promotes the development of oocytes into preimplantation embryos. Proc Natl Acad Sci USA.

[39] Kawamura K, Kumagai J, Sudo S, Chun SY, Pisarska M, Morita H (2004). Paracrine regulation of mammalian oocyte maturation and male germ cell survival. Proc Natl Acad Sci USA.

[40] Yuan YQ, Van Soom A, Leroy JL, Dewulf J, Van Zeveren A, de Kruif A (2005). Apoptosis in cumulus cells, but not in oocytes, may influence bovine embryonic developmental competence. Theriogenology.

[41] Horner VL, Wolfner MF (2008). Mechanical stimulation by osmotic and hydrostatic pressure activates Drosophila oocytes in vitro in a calcium-dependent manner. Dev Biol.

[42] Charras GT, Yarrow JC, Horton MA, Mahadevan L, Mitchison TJ (2005). Non-equilibration of hydrostatic pressure in blebbing cells. Nature.

